# DNA damage chemotherapeutic drugs suppress basal-like breast cancer growth by down-regulating the transcription of the FOXO1-KLF5 axis

**DOI:** 10.1016/j.gendis.2023.03.028

**Published:** 2023-05-02

**Authors:** Qiuxia Cui, Jian Sun, Jingping Yuan, Juanjuan Li, Chuanyu Yang, Guangshi Du, Chengang Zhou, Pu Qiu, Jidong Gao, Yuanqi Zhang, Dewei Jiang, Ceshi Chen

**Affiliations:** a Affiliated Hospital of Guangdong Medical University Science & Technology of China, Zhanjiang, Guangdong 524000, China; b Department of Breast Surgical Oncology, National Cancer Center/National Clinical Research Center for Cancer/Cancer Hospital and Shenzhen Hospital, Chinese Academy of Medical Sciences and Peking Union Medical College, Shenzhen, Guangdong 518116, China; c Key Laboratory of Animal Models and Human Disease Mechanisms of the Chinese Academy of Sciences and Yunnan Province, Kunming Institute of Zoology, Chinese Academy of Sciences, Kunming, Yunnan 650223, China; d Department of Pathology, Renmin Hospital of Wuhan University, Wuhan, Hubei 430060, China; e Department of Breast and Thyroid Surgery, Renmin Hospital of Wuhan University, Wuhan, Hubei 430060, China; f Translational Medicine Research Center of Guizhou Medical University, Guiyang, Guizhou 550025, China; g Department of Breast Surgical Oncology, National Cancer Center/National Clinical Research Center for Cancer/Cancer Hospital, Chinese Academy of Medical Sciences and Peking Union Medical College, Beijing 100021, China; h Yunnan Cancer Hospital and the Third Affiliated Hospital of Kunming Medical University and Yunnan Cancer Center, Kunming, Yunnan 650500, China; i Academy of Biomedical Engineering, Kunming Medical University, Kunming, Yunnan 650500, China

Most basal-like breast cancers (BLBCs) have a poor prognosis and high crossover with triple-negative breast cancers (TNBCs). However, approximately 50% of TNBC patients develop chemoresistance.^1^ Dexamethasone reportedly induces Krüppel-like factor 5 (KLF5) and can cause docetaxel and cisplatin resistance in TNBC. A super-enhancer can maintain the transcription of *KLF5*.^2^ Bromodomain-containing 4 (BRD4) is a transcriptional coactivator in super-enhancers, and the BRD4 inhibitor compound 870 could strongly inhibit KLF5 transcription.^2^ Forkhead box class O 1 (FOXO1) promotes chemoresistance to doxorubicin in breast cancer.^3^ FOXO1 has been shown to promote *SOX2* transcription, which plays a critical role in cancer stemness.^4^ In addition, FOXO1 promoted *KLF5* transcription in diabetic cardiomyopathy.^5^ However, the roles of FOXO1 and KLF5 in chemotherapy remain exclusive. In the present study, we examined the function of FOXO1 and the relationship between FOXO1 and KLF5 in BLBCs. We used epirubicin (EPI) and cisplatin (DDP) to treat BLBCs. The expression of FOXO1 and KLF5 was down-regulated, whereas KLF5 overexpression decreased the sensitivity of BLBCs to these drugs. FOXO1 regulated KLF5 in the BLBC chemotherapy response, and a novel therapeutic strategy targeting KLF5 and FOXO1 was proposed.

We analyzed breast cancer samples from public databases and found no notable differences in KLF5 expression between the residual disease (RD) and pathological complete response (pCR) groups after neoadjuvant chemotherapy in all breast cancer samples (Fig. 1A). However, KLF5 expression was higher in BLBC patients with RD than in those with pCR after neoadjuvant chemotherapy (Fig. 1B). Based on survival analysis, high KLF5 expression in patients receiving adjuvant chemotherapy was associated with poor overall survival (Fig. 1C) and disease-free survival (Fig. 1D).

**Figure 1:**
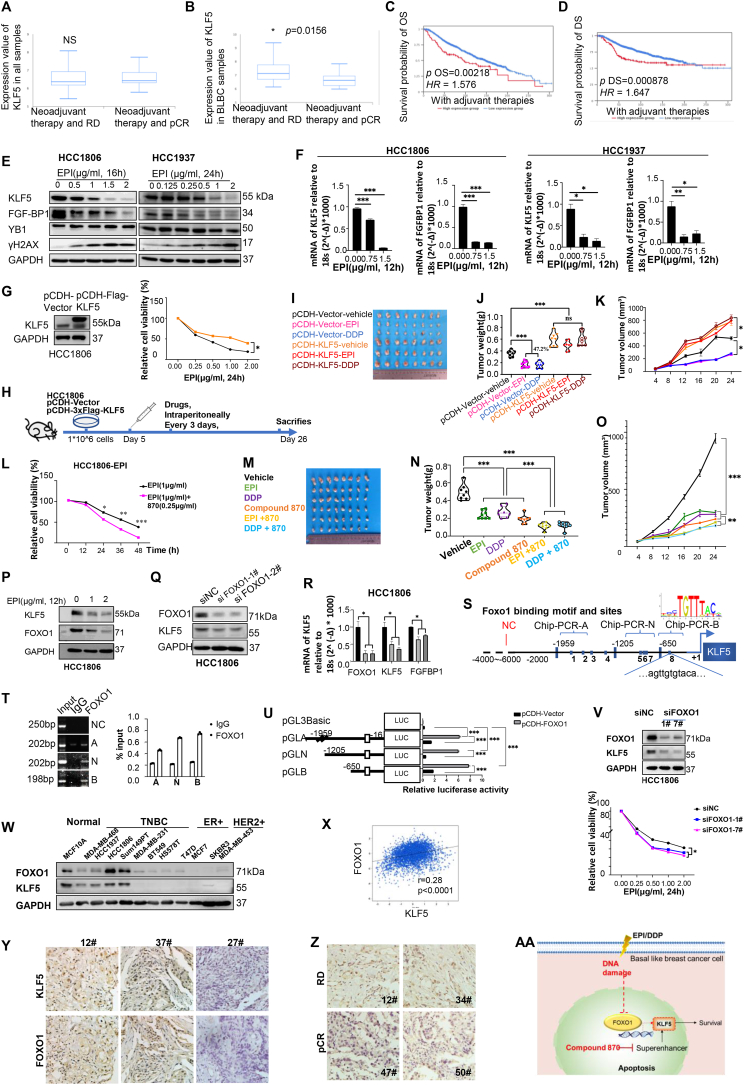
DNA-damaging drugs down-regulate KLF5 transcription by inhibiting FOXO1. **(A)** KLF5 expression in samples from all kinds of patients with breast cancer presenting RD and pCR after adjuvant chemotherapy. No significant difference was observed (*P* = 0.602). **(B)** In patients with triple-negative breast cancer receiving neoadjuvant chemotherapy, the level of KLF5 is significantly higher in the RD group than that in the pCR group (*P* = 0.0156). BLBC, basal-like breast cancer. The overall survival **(C)** and disease-free survival **(D)** in patients with high KLF5 expression are significantly worse than those in patients with low KLF5 expression. OS, overall survival; DS, disease-free survival. OS: *P* = 0.00218, HR = 1.576; DS: *P* = 0.000878, HR = 1.647). **(E, F)** HCC1806 and HCC1937 cells were treated with EPI for 24 h, and the expression of KLF5 and downstream genes of FGF-BP1 were detected by western blotting and real-time PCR. **(G)** HCC1806 and HCC1937 cells overexpressing KLF5 were treated with DDP and EPI for 24 h, and cell viability was detected by the SRB assay. **(H)** KLF5-overexpressing HCC1806 cells and control cells were transplanted into BALB/c nude mice (female, 6–8 weeks old). *n* = 4 in each group. EPI or DDP was administered intraperitoneally to tumors at day 6, every 4 days for 20 days. The tumor size **(I)**, tumor weight **(J)**, and tumor volume **(K)** were measured for each group. **(L)** HCC1806 cells were treated with chemotherapeutic EPI in combination with drugs that down-regulate KLF5 (compound 870) for 24 h, and cell activity was detected using the SRB assay. **(M**–**O)** HCC1806 cells were transplanted into BALB/c nude mice, female, 6–8 weeks old, *n* = 4 in each group. The tumors were treated with EPI or DDP, or combined with compound 870 on day 5 after injection, intraperitoneally, every 4 days for 20 days. The tumor size (M), tumor weight (N), and tumor volume (O) were measured in each group. **(P)** HCC1806 cells were treated with EPI for 12 h, and the down-regulation of KLF5 and FOXO1 was detected by western blotting. **(Q, R)** siRNA knockdown of FOXO1 decreases protein and mRNA levels of KLF5 in HCC1806 cells. **(S)** JASPAR prediction of binding sites of FOXO1 on the KLF5 promoter at *Homo sapiens* chromosome 13, GRCh38.p13, NC_000013.11:73052976-73054975. **(T)** HCC1806 cells were subjected to ChIP-PCR. **(U)** HEK293T cells transfected with pGL3-KLF5-promoter A (−1500 bp), N (−1000 bp), and B (−700 bp) (−553∼−542) were subjected to the dual luciferase assay. **(V)** siRNAs-mediated FOXO1-down-regulated HCC1806 cells were treated with EPI for 24 h, and cell viability was detected using the SRB assay. **(W)** The expression of FOXO1 and KLF5 in different breast cancer cell lines was measured using western blotting. **(X)** The co-expression of KLF5 and FOXO1 was analyzed in the TCGA database. **(Y)** The expression of KLF5 and FOXO1 in TNBC samples. 12# and 37# are two representatives of positive samples, and 27# is a representative of negative sample. **(Z)** The FOXO1 expression in samples developing RD or pCR after neoadjuvant chemotherapies. **(AA)** The working model of FOXO1 and KLF5 regulation in breast cancer cells treated with DNA damage drugs. ^∗^*P* < 0.05, ^∗∗^*P* < 0.01, ^∗∗∗^*P* < 0.001. ChIP-PCR, chromatin-immunoprecipitation-PCR; DDP, cisplatin; EP, epirubicin; FOXO1, Forkhead box class O 1; KLF5, Krüppel-like factor 5; pCR, pathological complete response; RD, residual disease; SRB, sulforhodamine B; TCGA, The Cancer Genome Atlas; TNBC, triple-negative breast cancer.

We treated the BLBC cell lines HCC1806 and HCC1937 with commonly used chemotherapeutic drugs DDP and EPI. The viabilities of HCC1806 and HCC1937 cells decreased in a dose-dependent manner (Fig. S1F, G). Given that both EPI and DDP are DNA-damaging drugs, we examined the levels of γH2AX, a biomarker of DNA damage. Both chemotherapeutic drugs could induce the accumulation of γH2AX in HCC1806 and HCC1937 cells. We observed that protein levels of KLF5 and its downstream target gene *FGF-BP1* were down-regulated in a dose- and time-dependent manner (Fig. 1E; Fig. S1A–C). Consistently, DDP and EPI significantly decreased *KLF5* and *FGF-BP1* mRNA levels (Fig. 1F; Fig. S1D, E). Relative primers and antibodies were shown in Table S1 and Table S2. However, other chemotherapeutic agents, including irinotecan (CPT-11) and camptothecin (CPT), did not impact KLF5 or FGF-BP1 protein expression (Fig. S1H–K). Y-box binding protein 1 (YB-1) is a master transcription factor upstream of KLF5. However, DDP and EPI did not alter YB-1 protein expression levels in BLBC cells (Fig. 1E; Fig. S1B).

To determine whether KLF5 contributes to resistance to chemotherapy, we stably overexpressed KLF5 in HCC1806 and HCC1937 cells. As shown in Figure 1G and Figure S2A and B, KLF5 overexpression conferred resistance to chemotherapeutic drugs in both cell lines. *In vivo*, this finding was validated by injecting KLF5-overexpressing HCC1806 cells into female nude mice (Fig. 1H). As expected, KLF5-overexpressing grafts grew faster than control grafts (Fig. 1I–K). More importantly, EPI and DDP showed no significant therapeutic effects on the KLF5-overexpressing tumors, whereas both drugs significantly suppressed tumor growth in the control group. The overexpression of KLF5 in tumor tissues was validated by western blotting (Fig. S2C), and the body weights of the mice were monitored (Fig. S2D). These results suggested that KLF5 expression contributes to chemoresistance in BLBC cells.

According to our previous study,^2^ the BRD4 inhibitor compound 870 could efficiently down-regulate KLF5 expression in BLBC cells. We treated HCC1806 and HCC1937 cells with compound 870 and found that these breast cancer cells were more sensitive to EPI and DDP (Fig. 1L; Fig. S3A–C). This finding was validated *in vivo* by injecting HCC1806 cells into nude mice. Combination treatment with compound 870 and EPI or DDP synergistically suppressed tumor growth (Fig. 1M−O).

To characterize the mechanism through which chemotherapeutic drugs down-regulate KLF5 expression in BLBC, we searched the literature and found that FOXO1 induces KLF5 transcription and causes oxidative stress in diabetic cardiomyopathy.^5^ In the Gene Expression Omnibus (GEO) datasets, both KLF5 and FOXO1 expression levels increased in lapatinib-resistant BT474 and EFM192A cells (GSE155341, Fig. S2E), as well as in tamoxifen-resistant MCF7 cells (GSE128460, Fig. S2F). In addition, FOXO1 and KLF5 expression levels were positively correlated according to bioinformatics analysis (Fig. S2G). TNBC samples exhibited higher FOXO1 expression than non-TNBC samples (Fig. S2H), and higher FOXO1 levels were correlated with poor prognosis (Fig. S2I).

We found that EPI and DDP could down-regulate FOXO1 and KLF5 expression in BLBC cells (Fig. 1P; Fig. S4A). Simultaneously, the mRNA level of *FOXO1* was down-regulated by EPI and DDP (Fig. S4B, C). To determine whether FOXO1 positively regulates KLF5 transcription in BLBC cells, we knocked down *FOXO1* in HCC1806 and HCC1937 cells. We found that both the protein and mRNA levels of *KLF5* were down-regulated (Fig. 1Q, R; Fig. S4D, E). However, immunoprecipitation (IP) revealed that KLF5 did not interact with FOXO1 (Fig. S4F, G).

We further predicted eight putative FOXO1 binding sites in the promoter region of *KLF5* according to the JASPAR database (https://jaspar.genereg.net/) (Fig. 1S). We confirmed the direct binding of FOXO1 to the *KLF5* promoter using chromatin-immunoprecipitation (ChIP)-PCR (Fig. 1T). According to the luciferase assay, the binding site was located in the region of −553 bp to ∼ −542 bp (Fig. 1U). According to the above results, FOXO1 could promote *KLF5* transcription by binding to the *KLF5* promoter. Therefore, we measured the sensitivity of HCC1806 and HCC1937 cells with down-regulated FOXO1 to chemotherapy (Fig. 1V; Fig. S5A, B). FOXO1 down-regulation promotes EPI sensitivity in HCC1806 and HCC1937 cells. CPT-11 and CPT did not inhibit the expression of KLF5/FGF-BP-1, which could be explained by the failure of the examined DNA-damaging chemotherapeutic drugs to down-regulate the expression of FOXO1.

Furthermore, we measured the expression of FOXO1 and KLF5 in different breast cancer cell lines and found that levels of FOXO1 were comparable to those of KLF5 (Fig. 1W). FOXO1 and KLF5 were correlated with each other, according to the public database (Fig. 1X) (*r* = 0.28, *P* < 0.0001). Furthermore, we measured the protein expression of FOXO1 and KLF5 in clinical TNBC samples treated with neoadjuvant chemotherapy using immunohistochemistry (IHC) (Fig. 1Y). Relative clinical information of these samples was shown in Table S3. The expression levels of FOXO1 and KLF5 were positively correlated in the TNBC samples (Table S4). Furthermore, we measured the FOXO1 expression in patients who achieved RD or pCR after neoadjuvant chemotherapy (Fig. 1Z). According to the statistical analysis, the RD group had higher FOXO1 levels than the pCR group, indicating that high FOXO1 levels could be correlated with TNBC patients who reached RD after neoadjuvant chemotherapy (Table S5). However, there was no correlation between KLF5 levels and TNBC patients who developed RD after neoadjuvant chemotherapy.

Herein, we found that FOXO1 enhanced KLF5 transcription by binding to the KLF5 gene promoter. KLF5 overexpression increased tumor growth and chemotherapeutic drug resistance. Therefore, we developed a novel combination strategy to improve the standard therapeutic regimen for BLBC by targeting KLF5 (Fig. 1AA).

## Ethics declaration

All animal experiments and clinical samples were approved by the relevant institutions.

## Author contributions

QC performed most experiments and drafted the manuscript. JS, CY, and CZ performed the other experiments. GD provided some advice on the project. JG provided advice regarding the revisions. CC and DJ designed this study. CC, DJ, and YZ supervised the study. YH and LJ provided relevant clinical samples for TNBC.

## Conflict of interests

The authors declare that they have no conflict of interests.

## Funding

This work was supported by the National Key R&D Program of China (No. 2020YFA0112300), the 10.13039/501100001809National Natural Science Foundation of China (No. 81830087, U2102203, 82173014, 81872414), the Yunnan Fundamental Research Projects (China) (No. 202101AS070050, 202001AW070018), major projects for fundamental research of Yunnan Province, China (No. 202201BC070002), 10.13039/501100002367CAS “Light of West China” Young Scholar Program, and Yunnan Revitalization Talent Support Program (China) (Yunling Shcolar Project and Young Talent Project), the Guangdong Foundation Committee for Basic and Applied Basic Research projects (China) (No. 2022A1515012420), the Guangdong Medical University Affiliated Hospital Competitive Clinical Study (China) (No. LCYJ2021B003), and the Guangdong Province Characteristic Innovation Projects (China) (No. 2021KTSCX037).

## References

[1] Foulkes W.D., Smith I.E., Reis-Filho J.S. (2010). Triple-negative breast cancer. N Engl J Med.

[2] Chen C.H., Yang N., Zhang Y. (2019). Inhibition of super enhancer downregulates the expression of KLF5 in basal-like breast cancers. Int J Biol Sci.

[3] van den Berg M.C.W., Burgering B.M.T. (2011). Integrating opposing signals toward Forkhead box O. Antioxidants Redox Signal.

[4] Yu J.M., Sun W., Wang Z.H. (2019). TRIB3 supports breast cancer stemness by suppressing FOXO1 degradation and enhancing SOX2 transcription. Nat Commun.

[5] Kyriazis I.D., Hoffman M., Gaignebet L. (2021). KLF5 is induced by FOXO1 and causes oxidative stress and diabetic cardiomyopathy. Circ Res.

