# Supplementary material for: DNA damage chemotherapeutic drugs suppress basal-like breast cancer growth by down-regulating the transcription of the FOXO1-KLF5 axis
Source: Genes Dis. 2023 May 2;11(1):91–4. doi: 10.1016/j.gendis.2023.03.028 (PMC10425834; doi:10.1016/j.gendis.2023.03.028)
Supplement: Multimedia component 1 [file mmc1.docx]

**Table S1. Primers**

| **Names** | **Sequence (5’-3’)** |
| --- | --- |
| FOXO1 | F: AAGAGCGTGCCCTACTTCAA |
|  | R: CTGTTGTTGTCCATGGATGC |
| KLF5 | F: ACACCAGACCGCAGCTCCA |
|  | R: TCCATTGCTGCTGTCTGATTTGTAG |
| FGFBP1 | F: CGTGTGCTCAGAACAAGGTG |
|  | R: TGAGCAGCCAGTAGGAGGAA |
| 18S | F: CCTGAGAAACGGCTACCACATC |
|  | R: GCCTCGAAAGAGTCCTGTATTG |
| **Plasmid primer sequence** |  |
| foxo1-f-ecori-bamhi-pCDH | F: CGAGCTCAAGCTTCGatggccgaggcgcctcaggtg |
|  | R: ATTATCTAGAGTCGCGtcagcctgacacccagctatgtgtcgttg |
| pGL3-k-p-B3-mut | F: CAGAGAAtcgacaTACAAACTGCGCGGCCGCCCTGCG |
|  | R: GCAGTTTGTAtgtcgaTTCTCTGACAGATTGGGCTGG |
| **ChIP-PCR primer sequence** |  |
| CHIP-PGLA-KLF5 | F: GGGCTGCTTGGTTAGAGAAA |
|  | R: ACAATCAAAAAGCCGCAAAC |
| CHIP-PGLN-KLF5 | F: AAAACGCTTGCCATCAAATC |
|  | R: GCGCTGAGGCTTTTAGAGAT |
| CHIP-PGLB-KLF5 | F: CCACCCTAATTCCCCCTCTT |
|  | R: CAGCCTATGAGCAGGGAGAG |
| **Oligo sequence of siRNA** |  |
| Control siRNA/ shRNA | CTTACGCTGAGTACTTCGA |
| KLF5siRNA/shRNA 5# | CGAUUACCCUGGUUGCACAtt |
| KLF5siRNA/shRNA 7# | GAUGUGAAAUGGAGAAGUAtt |
| KLF5siRNA/shRNA J# | AAGCUCACCUGAGGACUCAtt |
| FOXO1 siRNA 1# | AUUAUGACGAAUUGAAUUCUU |
| FOXO1 siRNA 2# | GGUGUGUGUGUGUUGUAUAAA |
| FOXO1 siRNA 7# | CCCAGAUGCCUAUACAAACdtdt |
| FOXO1 siRNA 8# | CTCAAATGCTAGTACTATTAGdtdt |

**Table S2. Antibodies**

| **Antibody** | **No. of Catalogue** | **Supplier** | **Applications** |
| --- | --- | --- | --- |
|  |  |  |  |
| Flag | F3165 | Sigma | WB |
| KLF5 | / | Home made | WB |
| KLF5 | AF3758 | R&D | ChIP-seq / ChIP-qPCR |
| GAPDH | sc-32233 | Santa | WB |
| YB-1 | A7704 | Abclonal | WB |
| YB-1 | sc-398340 | Santa | RIP |
| FOXO1 | 2880S | CST | WB/ ChIP-qPCR |
| FOXO1 | Ab52857 | Abcam | IHC |
| FGFBP1 | AF1593 | R&D | WB |
| Yh2ax |  |  |  |
| Rabbit (DA1E) mAb IgG XP® Isotype Control | 3900 | CST | ChIP/IP |

**Table S3. Clinical information for breast cancer patients receiving neoadjuvant chemotherapy**

| **Characteristics** | | |  | |  | **N (%)** |  |
| --- | --- | --- | --- | --- | --- | --- | --- |
| **Gender** | | |  |  | |  |  |
|  | Female | |  |  | | 72(100) |  |
| **Neoadjuvant effects** | | | | | |  |  |
|  | | RD |  |  | | 39(54.2) |  |
|  |  | pCR |  |  | | 33(45.8) |  |
| **Ages** | |  |  | | |  |  |
|  | | Years old | Mean ± SD (minimum to maximum) | | | 48.39±10.814 (22-70) |  |
| **Molecular subtypes** | | | | | |  |  |
|  | | Luminal A |  |  | | 1(1.4) |  |
|  |  | Luminal B |  |  | | 8(11.1) |  |
|  |  | HER2 (2+) * | |  | | 10(13.9) |  |
|  |  | **TNBC** | |  | | 53(73.6) |  |
|  | |  | **Histological subtypes** | | |  |  |
|  | |  |  | Invasive ductal | | 49(92.5) |  |
|  | |  |  | Invasive lobular | | 1(1.9) |  |
|  | |  |  | Breast medullary carcinoma | | 1(1.9) |  |
|  | |  |  | Breast apocrine carcinoma | | 1(1.9) |  |
|  | |  |  | Acinic cell carcinoma | | 1(1.9) |  |
|  | |  | **Invasion** | | |  |  |
|  | |  |  | none | | 41(77.4) |  |
|  | |  |  | Innervation | | 1(1.9) |  |
|  | |  |  | Blood vessel carcinoma embolus | | 9(17.0) |  |
|  | |  |  | Both | | 2(3.8) |  |
|  | |  | **Lymph node metastasis** | | |  |  |
|  | |  |  | <3 | | 44(83.0) |  |
|  | |  |  | ≥3 | | 9(17.0) |  |
|  | |  | **Neoadjuvant effects** | | |  |  |
|  | |  |  | RD | | 30(56.6) |  |
|  | |  |  | pCR | | 23(43.4) |  |

* Without Fisher measurement results, cannot identify the HER2 amplification.

**Table S4.** The correlation between KLF5 and FOXO1 expression in TNBC samples receiving adjuvant chemotherapy（Negative ≤0, Positive >0 ）

| KLF5 * FOXO1 * correlation in TNBC samples | | | | | | | | | |
| --- | --- | --- | --- | --- | --- | --- | --- | --- | --- |
|  | | | FOXO1 | | | | Total | X^2^ | p |
|  |  |  | Negative | | Positive | |  |  |  |
| KLF5 | Negative | 3 | | 1 | | 4 | | 7.791 | 0.005 |
|  | Positive | 6 | | 33 | | 39 | |  |  |
| Total | | 9 | | 34 | | 43 | |  |  |

**Table S5.** The correlation between neoadjuvant chemotherapy and FOXO1 expression in TNBC samples

| EFFECTS * FOXO1 * correlation in TNBC samples | | | | | | | | | |
| --- | --- | --- | --- | --- | --- | --- | --- | --- | --- |
|  | | | FOXO1 | | | | Total | X^2^ | p |
|  |  |  | ≤30% | | ＞30% | |  |  |  |
| Effects | RD | 8 | | 14 | | 22 | | 3.949 | 0.047 |
|  | pCR | 14 | | 7 | | 21 | |  |  |
| Total | | 22 | | 21 | | 43 | |  |  |


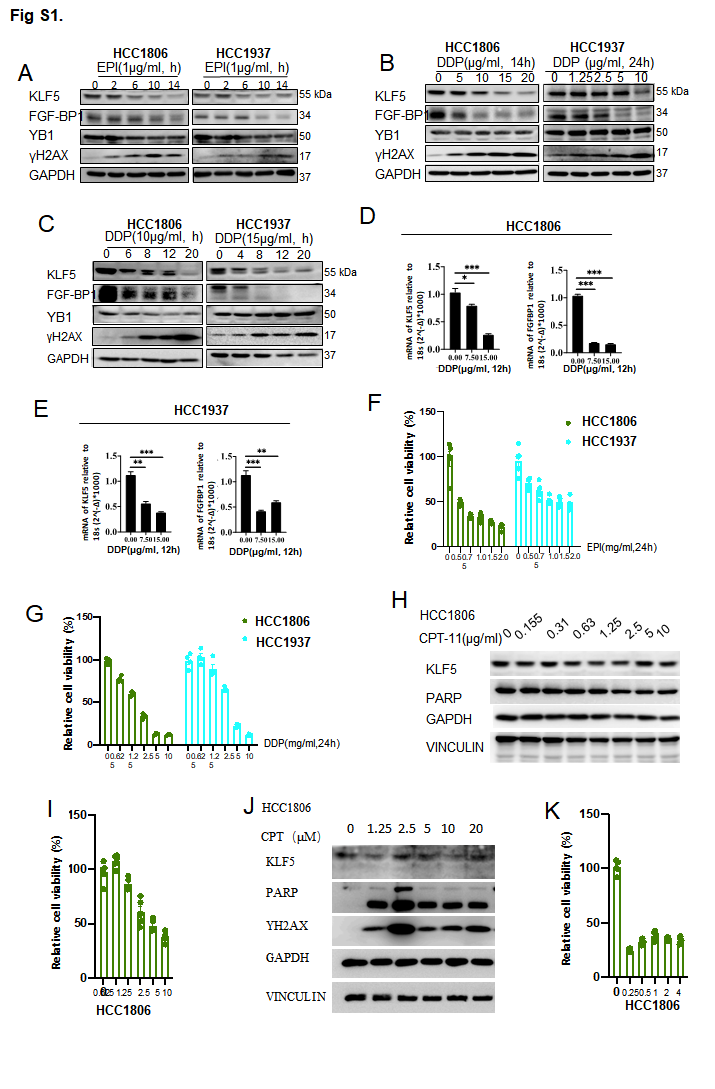


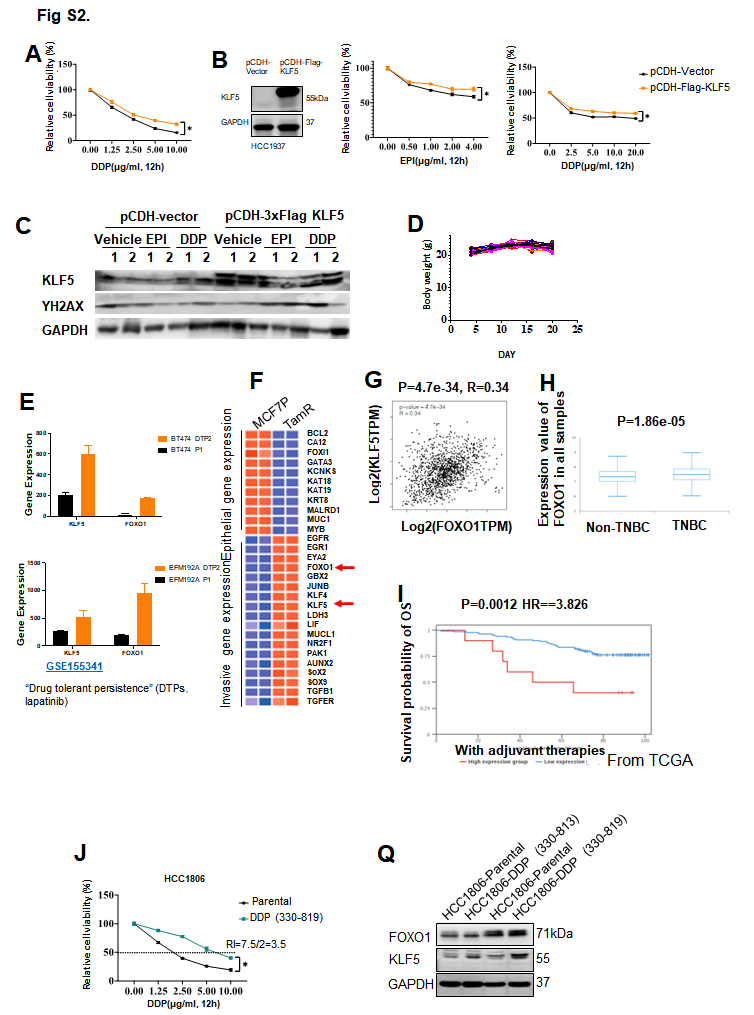


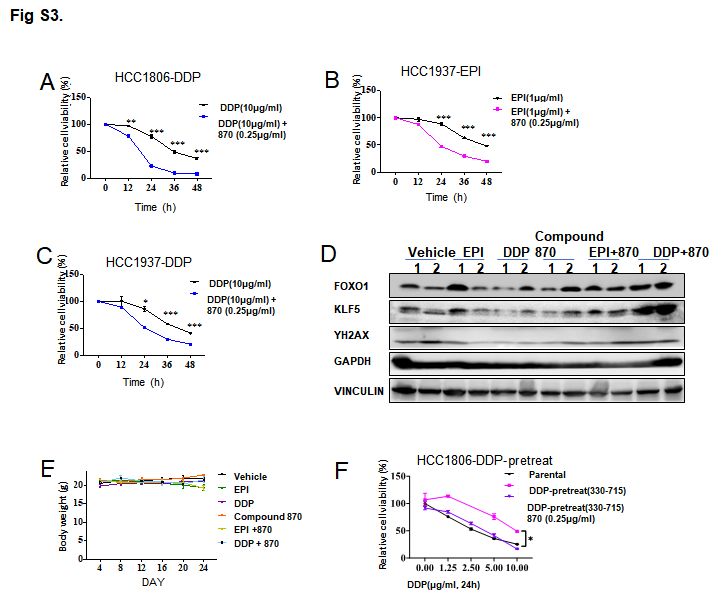


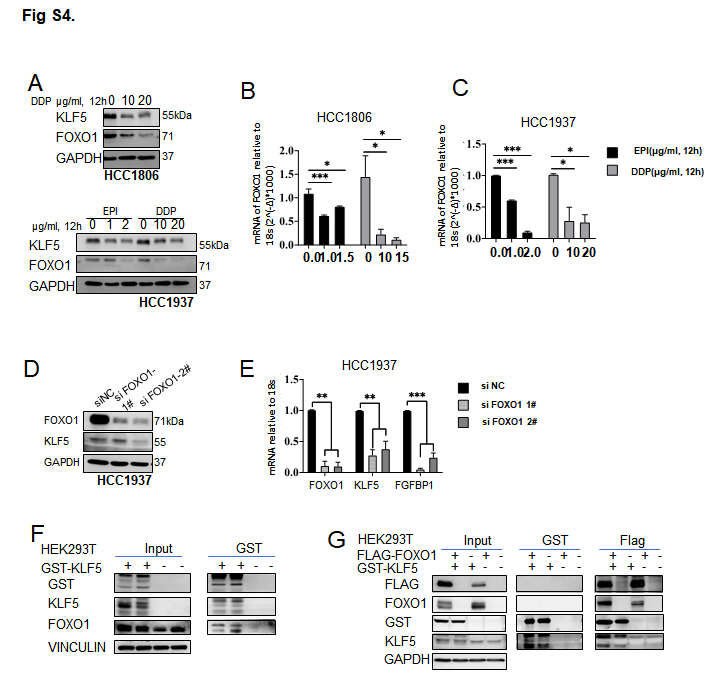


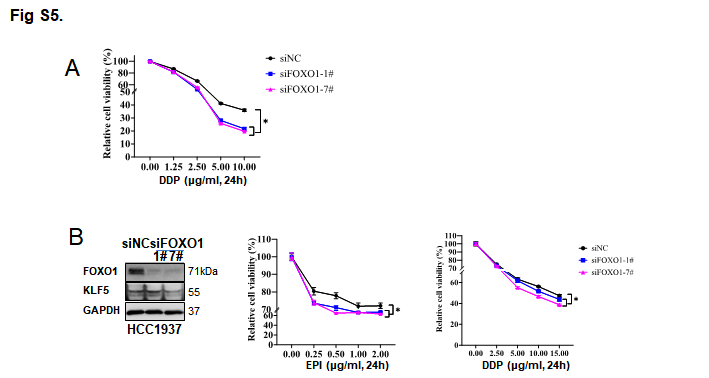


**Figure legend**

**Fig S1. KLF5 was downregulated by Epirubicin (EPI) and Cisplatin (DDP) in BLBC but not by other antitumor drugs**

**(A-C)** HCC1806 and HCC1937 cells were treated with DDP for 24 hours, the same time point of gradient concentration or the same concentration at gradient timespan, and the expression of KLF5 and downstream genes of FGF-BP1 were detected by Western blot and real-time PCR. **(D, E)** HCC1806 and HCC1937 cells were treated with EPI and cisplatin DDP for 24 hours, total RNA was collected, and the transcript levels of KLF5 and FGF-BP1 were measured by real-time PCR.

SRB about EPI(**F**) and DDP(**G**). Negative results by SRB about CPT-11 (**H, I**) and CPT (**J, K**)

(*, *p*<0.05; **, *p*<0.01; ***, *p*<0.001)

**Fig S2. KLF5 overexpression decreased the chemotherapy sensitivity of BLBC KLF5 was corelated with FOXO1**

**(A, B)** HCC1806 cells overexpressing KLF5 were treated with DDP and HCC1937 cells overexpressing KLF5 were treated with DDP and EPI for 24 hours, and cell viability was detected by SRB (paired *t* test, *, *p*<0.05; **, *p*<0.01). **(C)** The tumors tissues were measured by WB about the expression of KLF5, (D) mice body weight. (E) Gene expression of KLF5 and FOXO1 from GEO dataset (GSE 155341), DTPs resistant BT474 and EFM 192A cells; (lapatinib resistant). (F) Heatmap from GSE 128460, the tamoxifen resistant MCF7 cell showed upregulated invasive gene, including KLF5 and FOXO1.(G) The co-relationship of FOXO1 and KLF5 from TCGA database. (H) The higher level of FOXO1 in TNBC than non-TNBC samples. (I) Higher level of FOXO1 is correlated with poorer survival. (J and K) HCC1806 cells were pretreated with DDP at low concentration (1μg/ml) for about 4 months. The chemotherapeutic sensitivity was measured by SRB and the KLF5 expression was measured by WB.

**Fig S3. KLF5 inhibition enhances chemotherapeutic efficiency in vitro and in vivo.**

**(A)** HCC1806 cells were treated with chemotherapeutic DDP in combination with drugs that downregulate KLF5 (compound 870) for 24 hours, and cell activity was detected by SRB. (**B** and **C**) HCC1937 cells were treated with chemotherapeutic EPI and DDP in combination with drugs that downregulate KLF5 (compound 870) for 24 hours, and cell activity was detected by SRB (t test, *, *p*<0.05; **, *p*<0.01, ***, *p*<0.001). **(D)** The tumors tissues were measured by the WB (D) about the expression of KLF5 and FOXO1 and the mice body weight was monitored and visualized (**E**). (**F**) The chemotherapeutic sensitivity in DDP-pretreated HCC1806 cells was rescued by the combination with compound 870. (paired t-test, *, p<0.05)

**Fig S4. FOXO1 promotes the transcription of KLF5 in BLBC.**

**(A)** HCC1806 cells were treated with the chemotherapeutic drugs DDP, HCC1937 cells were treated with the chemotherapeutic drugs DDP and EPI for 12 hours, and the downregulation of KLF5 and FOXO1 was detected by WB **(A)** and qPCR **(B** and **C)**. **(D** and **E)** The protein and mRNA levels of KLF5 were decreased after siRNA knockdown of FOXO1 in HCC1937 cells. (**F** and **G**) IP results about FOXO1 and KLF5.

**Fig S5. Downregulation of FOXO1 increased the EPI sensitivity of HCC1806 and HCC1937 cells.**

**(A)** FOXO1-downregulated HCC1806 were treated with DDP for 24 hours and cell viability was detected by SRB. **(B)** HCC1937 cells by siRNAs were treated with EPI or DDP for 24 hours, and cell viability was detected by SRB (*t* test, *, *p*<0.05; **, *p*<0.01).

**Methods and materials**

*Cell culture*

MDA-MB-231 and HEK293T cells were grown in Dulbecco modified Eagle medium (Gibco, USA) containing 5% FBS. All cells were stored in a 5% CO2 incubator at 37 ° C.

BLBC cell lines, HCC1937 and HCC1806, were acquired from the American Type Culture Collection (ATCC, USA). The cells were cultured in the medium suggested by ATCC: Rosewell Park Memorial Institute-1640 (RPMI-1640) medium (Gibco, USA) with 5% fetal bovine serum (FBS, Gibco) for HCC1937 and HCC1806; Dulbecco’s modified Eagle’s medium (Gibco, USA) with 5% FBS for MDA-MB-231 and HEK293T cells. All cells were maintained in the condition of 5% CO_2_ at 37 °C.

*Plasmids, transfection and lentivirus*

The pCDH plasmid expressing KLF5 was described in our previous study[1]. The C-terminus of KLF5 was added with three Flag tags. The pCDH-Flag plasmid expressing FOXO1 was kindly provided by Prof. Ke Li [2] and the pCDH notag plasmid expressing FOXO1 was constructed. HCC1806 or HCC1937 cells with stable KLF5 or FOXO1 overexpression were prepared by lentiviral infection and selected using puromycin [3]. KLF5 and FOXO1 expression were confirmed by WB.

All plasmids and siRNAs were diluted in Opti-MEM (Gibco, USA) and transfected into cells by employing PEI (polyethylenimine, Plybiosciences) or Lipofectamine 2000 (Invitrogen) according to the manufacturer protocol.

For HEK293T packaging cells, plasmids were transfected with 1 μg plasmid: 4 μL PEI. The viral supernatant was obtained and added to recipient cell medium with 8 μg/mL polybrene for 24 hours. The medium was refreshed later and 1 μg/mL puromycin was added to the medium to select cell populations. All chemically synthesized siRNAs were purchased from RiboBio and transfected at a final concentration of 10 nM. The siRNA target sequences are provided in Supplementary Table 1. KLF5 was knocked down by siRNA in HCC1806 and HCC1937 cells [4].

*Western blot analysis*

RIPA buffer and a protease inhibitor cocktail (MCE, HY-K0010) were employed to get the cell lysis. After concentration quantification, 25 μg of total protein was utilized to perform SDS-PAGE and WB analysis. The primary antibodies and specific secondary antibodies were employed in the following steps, and detailed information about the antibodies was described in the Supplemental Table 2. Clarity Western ECL Substrate (Bio-Rad) was applied to obtain the results.

*Sulforhodamine B assays (SRB)*

Cells were seeded in 96-well plates and treated with different concentration of DDP or EPI for 24 hours. And the cell viability was measured following the standard protocol[5].

*Real-time PCR (RT-PCR)*

Total RNA was extracted from HCC1806 and HCC1937 after EPI or DDP treatment and reverse transcribed using the iScript™ cDNA Synthesis Kit. The target gene mRNA levels were quantified in triplicate by employing AceQ qPCR SYBR Green Master Mix (Vazyme, Q111-02) and gene expression analysis was quantified by using the 2^(-ΔΔCT) comparative method[6]. Simutaneous amplification of the target sequences (*FOXO1*, *KLF5*, *FGF-BP1*) together with the housekeeping gene *18S* ribosomal RNA (rRNA) was carried out essentially as previously described[7]. Primers are listed in Supplemental Table 1.

*Chromatin immunoprecipitation (ChIP)*

ChIP assays were performed using the HCC1806 cell line following the manufacturer’s instructions (Abcam, USA) with slight modifications described previously [8]. The DNA-protein complex from HCC1806 was incubated with anti-FOXO1 (CST, #2880s) and rabbit IgG (CST, #3900) antibodies and Protein A/G magnetic beads (MCE, HY-K0202) at 4 °C overnight. The chromosomal DNA was purified and analyzed by rourine or quantitative PCR. Primers for ChIP are presented in Supplementary Table 1.

*Dual luciferase reporter assay*

The *KLF5* promoter luciferase reporter plasmid was constructed in our previous work [9]. The *KLF5* promoter was cloned into the pGL3-BASIC plasmid (Promega, Madison, WI), including the plasmids of pGL3A (the longest one, 1944 bp), PGL3N (1309 bp) and PGL3B (754 bp) [9]. Plasmids of PGL3B-mut (binding site-8 mutant) were generated using the PCR-directed mutagenesis method. The primers are presented in Supplementary Table1. HEK293T cells (5 × 10^4^ cells per well) were seeded in 24-well plates and cultured for 16–24 hours. The cells were transfected with pCDH-3xFlag-FOXO1 and pGL3 luciferase reporter plasmids (both 300 ng/well) together with pCMV-Renilla control (10 ng/well) for 48-72 hours. Then the cells were collected and lysed in the lysis buffer (Promega) following the protocol. Each sample was checked in triplicate and the luciferase activities were measured through the luciferase assay reagent system (Promega).

*Animal models*

HCC1806 cells with stable KLF5 overexpression were prepared as described above. Twenty-four female nude mice (6 weeks old, BALB/c, SJA Laboratory Animal Co., Ltd, China) were randomly distributed into two groups (pCDH-Vector and pCDH-3xFlag-KLF5, each group contained 12 mice). HCC1806 cells (1 × 10^6^) were injected subcutaneously near the mammary fat pads of mice. Each 12-mouse group was randomly distributed into 3 groups, each containing 4 mice, and treated with vehicle, EPI (2 mg/kg) or DDP (2 mg/kg). The tumors were treated with vehicle, EPI or DDP at day 6 after injection, intraperitoneally, every 4 days, for 20 days. Tumor sizes were measured every four days using Vernier calipers. The tumor volumes were calculated as follows: tumor volume (mm^3^) = 0.5 × length × width^2^. The tumors were harvested and weighed when the mice were sacrificed on day 26. The animal experiment was approved by the Animal Ethics Committee of the Kunming Institute of Zoology, CAS.

Administration of the combination of BRD4 inhibitor (compound 870) with EPI or DDP in tumor-bearing mouse HCC1806 cells (1 × 10^6^) was injected subcutaneously close to the mammary fat pads of 24 female nude mice (6 to 7 weeks old). After 6 days, mice were randomly distributed into 6 groups. The mice were then treated with vehicle, EPI, DDP, compound 870, EPI plus compound 870, and DDP plus compound 870 by intraperitoneal injection every 4 days. The tumor volume and weight of mice were measured every other day and the tumors were harvested and weighed on day 26. All drugs were prepared in ddH_2_O.

*Clinical samples and immunohistochemistry*

The tissue slides of human breast cancer were provided by Renmin Hospital of Wuhan University and the clinical information is listed in Supplementary Table 3. Informed consents from all participants were obtained and the application of human specimens was approved by the Institutional Review Board of the Renmin Hospital of Wuhan University and the Affiliated Hospital of Guangdong Medical University.

*Gene expression analysis from the public database*

All microarray data employed in this study were available in the Gene Expression Omnibus (GEO) repository, with the accession codes GSE 155341, GSE 128460 and GSE 20271_GPL98. The survival analysis of FOXO1 and KLF5 expression with overall survival and disease-free survival in the breast cancer dataset was obtained from The Cancer Genome Atlas (TCGA) and Metabric.

*Statistical analysis*

SRB assay and RT PCR significance were calculated by paired *t* test analysis. Tumor growth rate and the analysis of tumor weight significance were calculated by one-way ANOVA and unpaired Student’s *t* test. The association of FOXO1 and KLF5 with RD was assessed by chi-square test analysis. Data are shown as the mean ± SEM using GraphPad Prism 6 or SPSS 17.0 (SPSS, Inc.).

**References**

1. Chen CH, Yang N, Zhang Y, Ding J, Zhang W, Liu R, Liu W, & Chen C: (2019). Inhibition of super enhancer downregulates the expression of KLF5 in basal-like breast cancers. *Int J Biol Sci*, *15*(8):1733-1742. <https://doi.org/10.7150/ijbs.35138>

2. Yu JM, Sun W, Wang ZH, Liang X, Hua F, Li K, Lv XX, Zhang XW, Liu YY, Yu JJ, Liu SS, Shang S, Wang F, Yang ZN, Zhao CX, Hou XY, Li PP, Huang B, Cui B, & Hu ZW: (2019). TRIB3 supports breast cancer stemness by suppressing FOXO1 degradation and enhancing SOX2 transcription. *Nat Commun*, *10*(1):5720. <https://doi.org/10.1038/s41467-019-13700-6>

3. Jiang D, Qiu T, Peng J, Li S, Tala, Ren W, Yang C, Wen Y, Chen CH, Sun J, Wu Y, Liu R, Zhou J, Wu K, Liu W, Mao X, Zhou Z, & Chen C: (2022). YB-1 is a positive regulator of KLF5 transcription factor in basal-like breast cancer. *Cell Death Differ*, *29*(6):1283-1295. <https://doi.org/10.1038/s41418-021-00920-x>

4. Du G, Sun J, Li Z, Zhang Q, Liu W, Yang C, Zhao P, Wang X, Yin Q, Luo Y, Song J, Wen Y, Wang H, Chen CH, Hu G, Zhou Z, Mao X, Liu W, Liu Z, Jiang D, & Chen C: (2022). A feedforward circuit between KLF5 and lncRNA KPRT4 contributes to basal-like breast cancer. *Cancer Lett*, *534*:215618. <https://doi.org/10.1016/j.canlet.2022.215618>

5. Vichai V, & Kirtikara K: (2006). Sulforhodamine B colorimetric assay for cytotoxicity screening. *Nat Protoc*, *1*(3):1112-1116. <https://doi.org/10.1038/nprot.2006.179>

6. van den Berg MC, & Burgering BM: (2011). Integrating opposing signals toward Forkhead box O. *Antioxid Redox Signal*, *14*(4):607-621. <https://doi.org/10.1089/ars.2010.3415>

7. Coomans de Brachene A, & Demoulin JB: (2016). FOXO transcription factors in cancer development and therapy. *Cell Mol Life Sci*, *73*(6):1159-1172. <https://doi.org/10.1007/s00018-015-2112-y>

8. Li F, Liang H, You H, Xiao J, Xia H, Chen X, Huang M, Cheng Z, Yang C, Liu W, Zhang H, Zeng L, Wu Y, Ge F, Li Z, Zhou W, Wen Y, Zhou Z, Liu R, Jiang D, Xie N, Liang B, Liu Z, Kong Y, & Chen C: (2022). Targeting HECTD3-IKKalpha axis inhibits inflammation-related metastasis. *Signal Transduct Target Ther*, *7*(1):264. <https://doi.org/10.1038/s41392-022-01057-0>

9. Chen C, Zhou Y, Zhou Z, Sun X, Otto KB, Uht RM, & Dong JT: (2004). Regulation of KLF5 involves the Sp1 transcription factor in human epithelial cells. *Gene*, *330*:133-142. <https://doi.org/10.1016/j.gene.2004.01.014>
