# Supplementary material for: DNA damage chemotherapeutic drugs suppress basal-like breast cancer growth by down-regulating the transcription of the FOXO1-KLF5 axis
Source: Genes Dis. 2023 May 2;11(1):91–4. doi: 10.1016/j.gendis.2023.03.028 (PMC10425834; doi:10.1016/j.gendis.2023.03.028)
Supplement: Multimedia component 2 [file mmc2.pdf]

打印该邮件　　关闭该页

Order delivery: Language Editing Express - Order reference ASLEEX0406395

发件人：webshop_support@elsevier.com
收件人：cherishcqx@sina.cn
日　期：2023年03月21日 15点55分

ELSEVIER

Your edited document is ready

Dear Qiuxia Cui,

Your edited document is now ready, please save the file to your desktop. The download link will expire after 6 months.

Order reference: ASLEEX0406395
Language Editing Express

Edited Document

DNA-damage-chemotherapeutic-drugs-suppress-basal-like-breast-cancer-growth-through-downregulating-the-transcription-of-FOXO1-KLF5-axis-1679067593594.docx　Save file to desktop

---

Please note:
If you make any alterations to your manuscript after we return it to you and your manuscript is rejected by a journal for language errors, you cannot return it to us for free re-editing. However if you do not alter your manuscript that we have edited and it is rejected by a journal on purely English Language grounds, you can receive either a full refund or have your message re-edited free of charge.

We wish you the best of luck with your article and thank you for choosing Elsevier Language Editing Services

If you have any other questions, please contact us at webshop_support@elsevier.com

Kind regards
Elsevier

© 2022 Elsevier Ltd | Privacy Policy | Privacy Policy http://www.elsevier.com/privacypolicy
Elsevier Limited, The Boulevard, Langford Lane, Kidlington, Oxford, OX5 1GB, United Kingdom, Registration No. 1982084. This e-mail has been sent to you from Elsevier Ltd.

ELSEVIER

RELX Group™
